# Mannose-Binding Lectin Regulates Host Resistance and Pathology during Experimental Infection with *Trypanosoma cruzi*

**DOI:** 10.1371/journal.pone.0047835

**Published:** 2012-11-06

**Authors:** Antonio Gigliotti Rothfuchs, Ester Roffê, Amanda Gibson, Allen W. Cheever, R. Alan B. Ezekowitz, Kazue Takahashi, Mario Steindel, Alan Sher, André Báfica

**Affiliations:** 1 Immunobiology Section, Laboratory of Parasitic Diseases, National Institute of Allergy and Infectious Diseases, National Institutes of Health, Bethesda, Maryland, United States of America; 2 Molecular Signaling Section, Laboratory of Molecular Immunology, National Institute of Allergy and Infectious Diseases, National Institutes of Health, Bethesda, Maryland, United States of America; 3 Biomedical Research Institute, Rockville, Maryland, United States of America; 4 Laboratory of Developmental Immunology, Department of Pediatrics, Massachusetts General Hospital, Harvard Medical School, Boston, Massachusetts, United States of America; 5 Department of Microbiology, Tumor and Cell Biology (MTC), Karolinska Institutet, Stockholm, Sweden; 6 Department of Microbiology, Immunology and Parasitology, Federal University of Santa Catarina, Florianópolis, Santa Catarina, Brazil; National Institutes of Health, United States of America

## Abstract

Mannose-binding lectin (MBL) is a humoral pattern-recognition molecule important for host defense. Although recent genetic studies suggest an involvement of MBL/MASP2-associated pathways in Chagas’ disease, it is currently unknown whether MBL plays a role in host resistance to the intracellular protozoan *Trypanosoma cruzi*, the causative agent of Chagas’ disease. In this study we employed MBL^−/−^ mice to assess the role of MBL in resistance to experimental infection with *T. cruzi*. *T. cruzi* infection enhanced tissue expression of MBL both at the mRNA and protein level. Similarly, symptomatic acute Chagas’ disease patients displayed increased serum concentrations of MBL compared to patients with indeterminate, asymptomatic forms of the disease. Furthermore, increased parasite loads in the blood and/or tissue were observed in MBL^−/−^ mice compared to WT controls. This was associated with reduced systemic levels of IL-12/23p40 in MBL^−/−^ mice. Importantly, MBL^−/−^ mice infected with a cardiotropic strain of *T. cruzi* displayed increased myocarditis and cardiac fibrosis compared to WT controls. The latter was accompanied by elevated hydroxyproline content and mRNA levels of collagen-1 and -6 in the heart. These observations point to a previously unappreciated role for MBL in regulating host resistance and cardiac inflammation during infection with a major human pathogen.

## Introduction

Collectins, with their collagenous backbone and globular carbohydrate-recognition domains, are major players in innate immunity. Mannose-binding lectin (MBL) is an important member of the collectin family and functions as a humoral pattern-recognition receptor (PRR) [1]. It is a serum opsonin that binds to microbial cell-wall saccharides present in various organisms, including gram-positive and -negative bacteria, yeast, protozoan parasites and viruses, and initiates the lectin pathway of complement activation [1]. A seminal study found that low MBL levels account for a common defect in opsonization of yeast in patients prone to recurrent infections [2]. This discovery subsequently led to a vast array of clinical studies associating MBL deficiency with susceptibility to infection [3]. Human genetic studies have since established a relationship between MBL allelic variants, serum levels of MBL and susceptibility to infection [4].

Studies on the biology of MBL and its role in infection have been greatly facilitated by the recent generation of gene-targeted mice lacking functional MBL [5]. Mice present two functional MBL proteins, MBL-A and MBL-C, while humans display only one [6]. The two murine forms are highly homologous to human MBL and both bind to carbohydrate surfaces and activate complement [7]. Mice deficient in both MBL-A and MBL-C and consequently lacking all circulating MBL (MBL^−/−^ mice) have been instrumental in unraveling a role for this molecule in host resistance to *Staphylococcus aureus* [5], [8], Herpes simplex virus-2 [9] and *Candida albicans* [10]. *In vitro* studies on *S. aureus* also revealed a role for MBL in modulating infection-induced cytokine and chemokine responses [11]. Moreover, isolated reports implicate MBL in regulating inflammation during post-burn injury [12], acute septic peritonitis [13] and kidney ischemia reperfusion injury [14]. However, there is a paucity of information regarding the role of MBL during protozoan infections and its inflammatory sequelae.

*Trypanosoma cruzi* is an intracellular protozoan parasite. It is the causative agent of Chagas’ disease, which affects roughly 17 million people throughout Central and South America [15]. Host control of this parasite is strongly coupled to cell-mediated, adaptive immune responses, in particular to T cells producing interferon (IFN)-γ [16]. A role for innate immunity in triggering such responses is evident from defects in adaptive immunity and host resistance to *T. cruzi* in mice with selective ablation of toll-like receptors (TLR) or the TLR adaptor molecule MyD88 [17], [18]. The contribution of humoral responses to protection against *T. cruzi* has also been studied. In this context, complement has been shown to bind to the surface of trypomastigotes, the blood-stage, infectious form of *T. cruzi,* but this form of the parasite demonstrates significant resistance to direct complement-mediated lysis [19]. Thus, while MBL has been shown to bind directly to *T. cruzi* trypomastigotes or their surface proteins *in vitro* [20], [21], activation of the lectin pathway is nevertheless inhibited [21]. Importantly, single nucleotide polymorphism (SNP) studies in humans indicate a possible function of MBL or MBL-associated pathways such as MASP2 in regulating *T. cruzi*-induced cardiomyopathy [22], [23]. In addition to complement, it is also possible that MBL could influence heart inflammation through an indirect mechanism. Regardless, how MBL regulates host responses to *T. cruzi* remains to be investigated *in vivo*.

In the present study, we report that MBL^−/−^ mice display a moderate increase in susceptibility to infection with both reticulo- and cardiotropic strains of *T. cruzi* accompanied by reduced systemic levels of IL-12/23p40. In addition, MBL^−/−^ mice exhibit increased inflammatory responses in the heart following infection with the cardiotropic strain of the parasite. Our data suggest that MBL is part of an important humoral pattern recognition system involved in host protective responses that help contain parasite replication *in situ* and regulate immunopathology.

## Results

### MBL is Induced Following *T. cruzi* Infection

**Figure 1:**
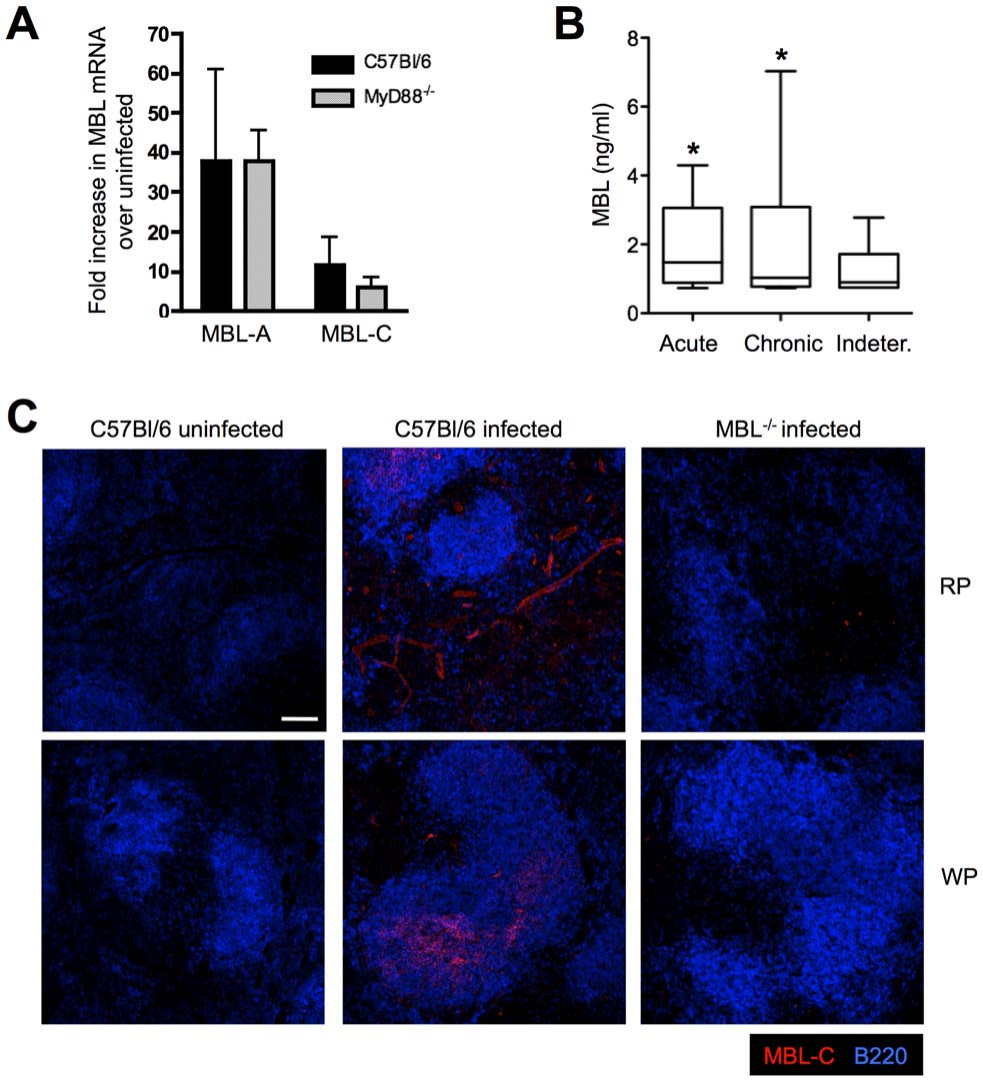
*T. cruzi* infection induces MBL expression *in vivo*. WT and MyD88^−/−^ mice were infected with trypomastigotes of the Y strain of *T. cruzi* and 9 days after infection, MBL-A and MBL-C mRNA accumulation was determined in spleens from infected and uninfected animals by real-time PCR (A). Detection of MBL-C protein was also performed by immunofluorescence microscopy in splenic sections obtained from WT mice infected as above (B). Micrograph shows MBL-C (red) and B220 (blue) staining in both the red (RP) and white pulp (WP). Scale bar, 100 µm. Infected MBL^−/−^ spleens were included as a control. All panels shown are representative of 2 independent experiments using 3 to 4 mice per group. Bars indicate the standard error of the mean (SEM). Sera from patients with acute, chronic or indeterminate Chagas’ disease were assayed for MBL as described in Material and Methods (C). *, Indicates statistically significant differences between acute vs indeterminate Chagas’ disease groups.

Previous studies have reported increased expression of MBL in response to inflammatory stimuli such as LPS or azocasein [24], [25] but less is known regarding its induction in response to live organisms. To investigate whether MBL is up-regulated during *T. cruzi* infection *in vivo*, mRNA expression of this lectin was assessed following parasite infection. Increased mRNA accumulation of both MBL-A and MBL-C was observed in spleen after i.p. infection with 1000 trypomastigotes of the reticulotropic Y strain of *T. cruzi*, with MBL-A induction being higher than MBL-C (Figure 1A). The observed increase in MBL mRNA in the spleen from parasite-infected mice was consistent with immunofluorescence staining for MBL-C in the same tissue, where MBL expression localized to both red and white pulp (Figure 1B). Interestingly, Chagas’ disease patients with acute symptoms displayed increased quantities of MBL protein in the serum when compared to indeterminate, asymptomatic Chagas’ disease subjects (Figure 1C, *p = 0.0266). Although the TLR-adaptor MyD88 is a major regulator of innate and adaptive immune responses against *T. cruzi* [17], [18], parasite-triggered MBL mRNA expression appeared to be independent of MyD88 (Figure 1A). Altogether, these data suggest that acute infection with *T. cruzi* enhances expression of MBL in humans as well as in mice.

### MBL^−/−^ Mice Display Impaired Resistance to Y strain *T. cruzi* Infection Accompanied by a Reduction in IL-12/23p40 Production

**Figure 2:**
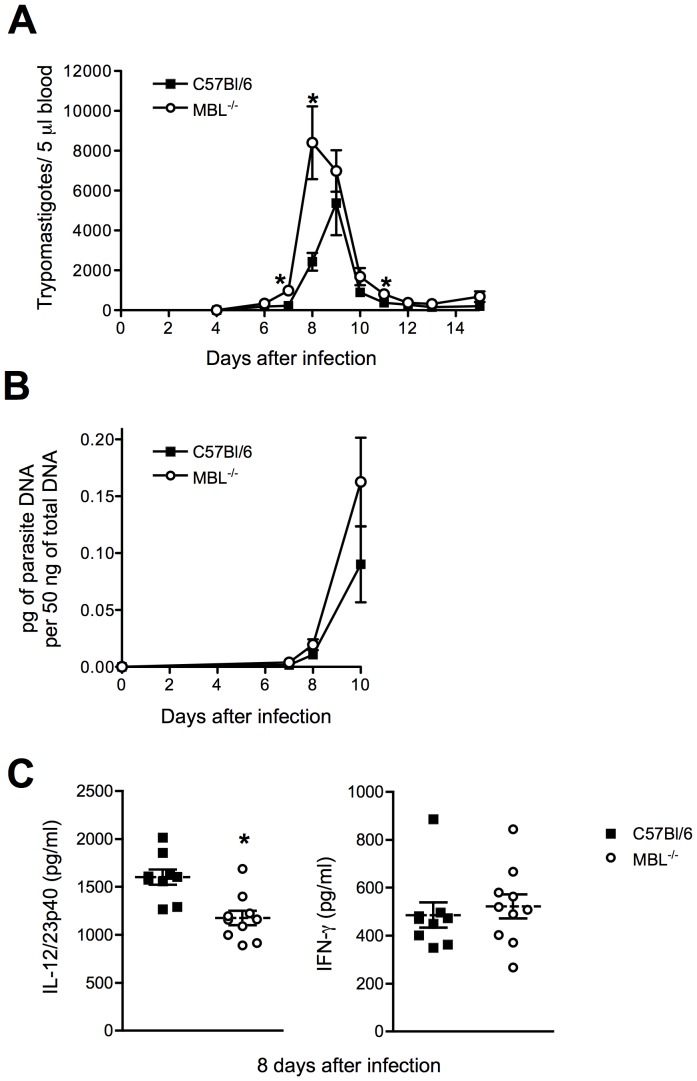
Role of MBL in host resistance to *T. cruzi* Y strain infection. WT or MBL^−/−^ mice were infected with trypomastigotes of the reticulotropic, Y strain of *T. cruzi* and monitored daily for parasitemia and mortality (A). Symbols represent the mean of 10 animals per group. At the indicated time points after infection, levels of parasite DNA in the spleen were quantified by real-time PCR (B). Serum levels of IL-12/23p40 and IFN-γ were determined by ELISA 8 days after infection (C). Each symbol in (B) and (C) corresponds to an individual animal, dashed lines the mean of each group and solid lines the standard error of the mean (SEM). The experiments shown are representative of at least two performed. *, Indicates statistically significant differences between groups.

Given the increased expression of MBL during infection, we investigated the role of this molecule in host resistance to *T. cruzi*. WT and MBL^−/−^ animals were infected with *T. cruzi* Y strain and followed for parasitemia and tissue parasite loads. Interestingly, MBL^−/−^ animals presented an increase in parasite numbers in the blood in comparison to WT mice (Figure 2A). Statistically significant differences in *T. cruzi* DNA load were not observed between WT and MBL^−/−^ mice although there was a tendency of higher parasite loads in MBL^−/−^ mice (Figure 2B). Reduced serum levels of IL-12/23p40 but not of IFN-γ were evident in infected MBL^−/−^ mice (Figure 2C). Consistent with normal production of IFN-γ, mortality was not observed in MBL-deficient animals.

### Increased Parasite Load, Cardiomyopathy and Fibrosis in MBL^−/−^ Mice Infected with *T. cruzi* Colombiana Strain

**Figure 3:**
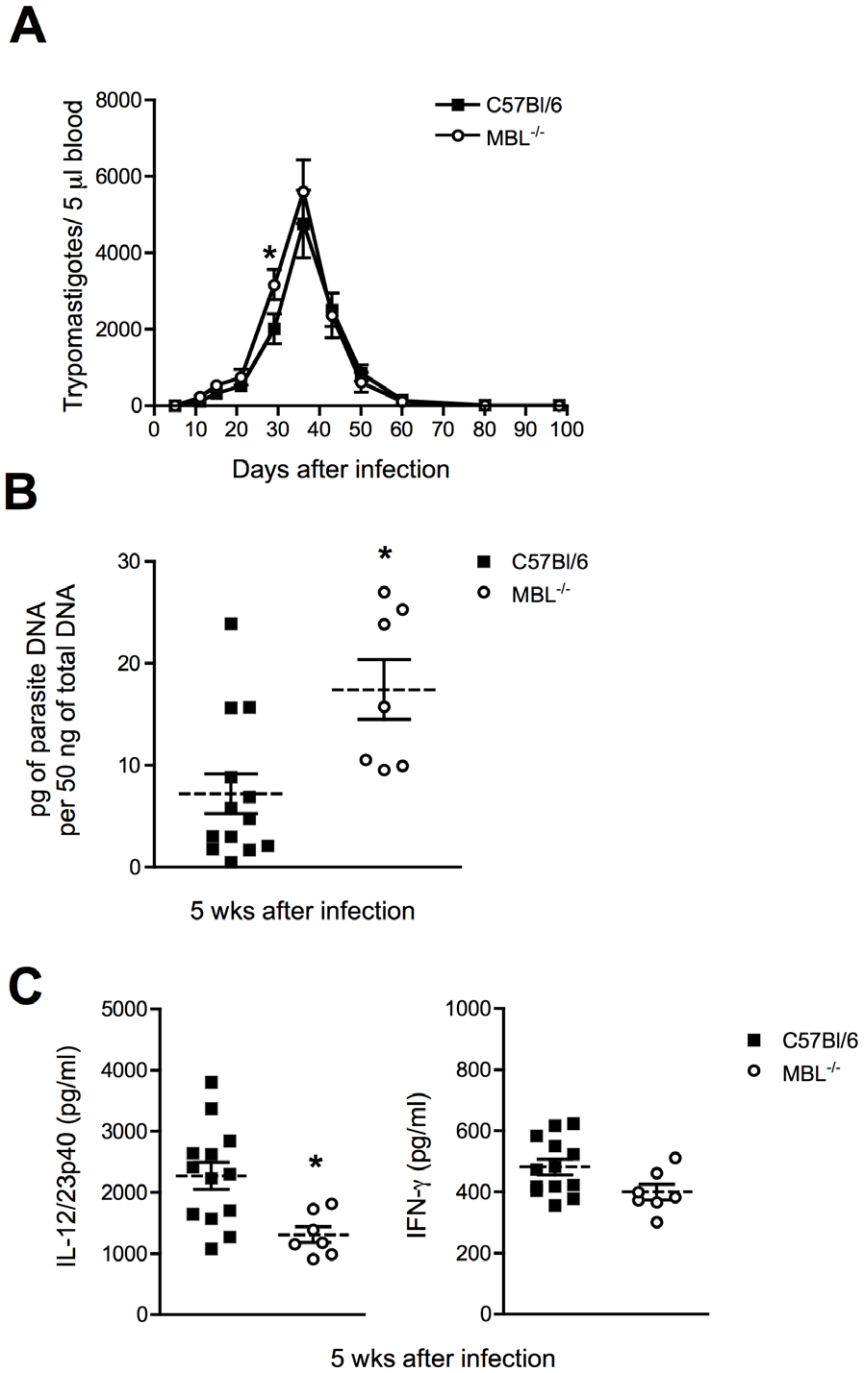
Role of MBL in host resistance to *T. cruzi* Colombiana strain infection. WT or MBL^−/−^ mice were infected with trypomastigotes of the cardiotropic, Colombiana strain of *T. cruzi* and monitored weekly for parasitemia and mortality (A). Symbols represent the mean of 9 to 10 animals per group. 5 wks after infection, levels of parasite DNA in the heart were quantified by real-time PCR (B) and serum levels of IL-12/23p40 and IFN-γ determined by ELISA (C). Each symbol in (B) and (C) corresponds to an individual animal, dashed lines the mean of each group and solid lines the standard error of the mean (SEM). The experiments shown are representative of two performed. *, Indicates statistically significant differences between groups.

Although experimental infection with the Y strain leads to only minor cardiac involvement [26], MBL^−/−^ mice displayed a higher number of amastigote nests in that tissue (data not shown). This suggested a potential involvement of MBL in regulating parasite replication in the heart. To address this and the role of MBL in cardiac inflammation triggered by *T. cruzi*, we infected WT and MBL^−/−^ mice with the cardiotropic Colombiana strain of *T. cruzi*. This strain is known to induce significant heart pathology in experimental animals [27]. While *T. cruzi* Colombiana-infected MBL^−/−^ animals displayed similar parasitemia to infected WT controls (Figure 3A), parasite loads in the hearts of MBL^−/−^ mice were elevated compared to WT controls at the peak of acute infection (Figure 3B). Consistent with the results observed during infection with the Y strain of the parasite, *T. cruzi* Colombiana-infected MBL^−/−^ mice displayed reduced systemic levels of IL-12/23p40 but not IFN-γ (Figure 3C). There was a tendency for increased mortality in MBL^−/−^ mice compared to infected WT controls, but this difference was not statistically significant (data not shown).

**Figure 4:**
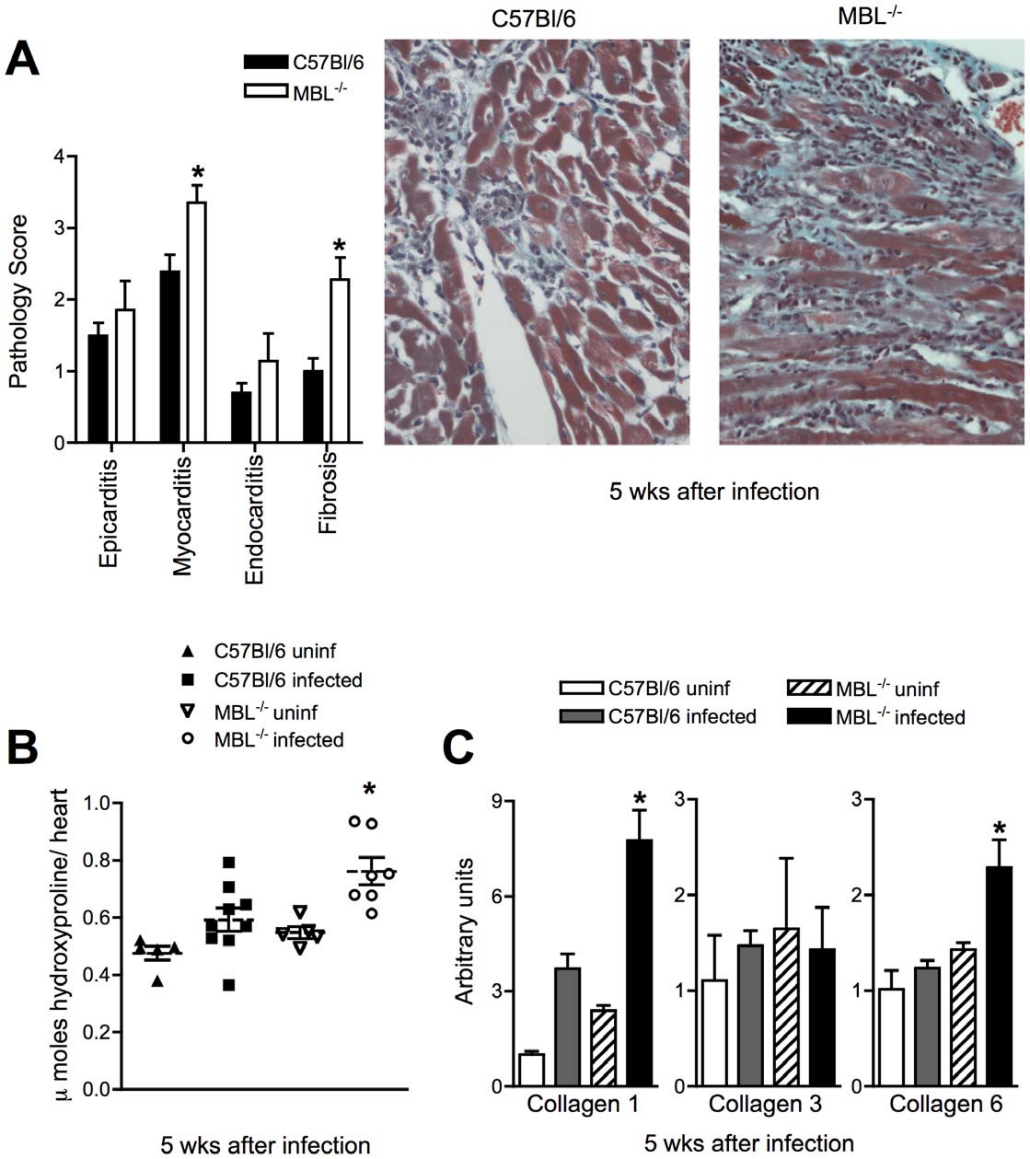
Enhanced cardiac pathology and fibrosis in MBL^−/−^ infected with *T.* *cruzi* Colombiana strain. WT and MBL^−/−^ mice were infected with trypomastigotes of the Colombiana strain of *T. cruzi* and hearts from these animals obtained 5 weeks after infection. Hearts were formalin-fixed, paraffin embedded, stained with H&E or Gomori’s trichrome and scored for pathology and fibrosis, respectively, as described under Materials and Methods (A). Right panels show micrographs of Gomori’s trichrome staining for each group. Bars indicate the standard error of the mean (SEM). Uninfected, or WT and MBL^−/−^ mice infected as in (A) were harvested 5 wks later for determination of hydroxyproline content in total heart (B). Each symbol corresponds to an individual animal, dashed lines represent the mean of each group and solid lines the standard error of the mean (SEM). Heart samples from uninfected controls or from WT and MBL^−/−^ mice infected as in (A) were obtained and mRNA accumulation of Collagen-1, -3 and -6 determined by real-time PCR (C). The experiments shown are representative of two performed. *, Indicates statistically significant differences between infected WT and MBL^−/−^ groups.

Myocarditis and cardiac fibrosis are hallmarks of the pathology observed in patients suffering from Chagas’ disease and in mice chronically infected with *T.* *cruzi* Colombiana. Such pathology lead to arrhythmias and heart failure in Chagas’ patients [28]. To assess the degree of cardiac disease in MBL^−/−^ mice chronically infected with *T.* *cruzi*, we performed histopathological analysis of hearts from Colombiana-infected WT and MBL^−/−^ mice. We found a major increase in both myocarditis and fibrosis in the heart of MBL^−/−^ mice 5 weeks after infection (Figure 4A). Myocarditis and fibrosis were mostly sub-epicardial, in the third or fourth of the myocardium nearest the heart surface, in agreement with previous histological data [27].

Augmented fibrosis in MBL^−/−^ mice was further corroborated by increased hydroxyproline content as well as Collagen-1 and -6 mRNA in the hearts from infected MBL^−/−^ but not WT animals (Figure 4B–C). Based on these and the above observations we conclude that MBL is an important regulator of heart inflammatory responses to *T. cruzi*.

## Discussion

A series of *in vivo* studies have recently implicated MBL in host control of bacterial, viral and fungal infections but the importance of MBL in host resistance to protozoa remains uncharacterized. In this study we addressed the role of MBL during experimental infection with reticulo- and cardiotropic strains of the protozoan parasite *Trypanosoma cruzi*. Our findings reveal that *T. cruzi* infection induces expression of both MBL-A and MBL-C through a MyD88-independent pathway and that MBL deficiency leads to a moderate increase in susceptibility to *T. cruzi*. The latter observation was associated with augmented tissue pathology and fibrosis. This is to our knowledge the first indication that MBL is important in mediating host resistance and in regulating inflammatory responses to an intracellular protozoan pathogen *in vivo*.

The liver is a major site of MBL synthesis under both steady-state and inflammatory conditions [24]. Detection of MBL-A and MBL-C has also been reported in various other tissues in the absence of exogenous stimuli [29], [30], but less is known regarding the extrahepatic expression of MBL during inflammation and infection. We observed increased expression of MBL-A and MBL-C in the spleen following live challenge with *T. cruzi*. Contrary to previous observations showing that MBL was present primarily in the marginal zone of naïve spleen [30], during *T. cruzi* infection we found MBL-C expression localized not only in the white pulp but also decorating vessels within the red pulp.

During *T. cruzi* infection, TLR triggering is important for production of IL-12/23p40, the ensuing Th1 response and consequently, host control of infection [31]. Interestingly, we found reduced levels of systemic IL-12/23p40 in *T. cruzi*-infected MBL^−/−^ mice. However, in spite of a higher parasite burden, MBL^−/−^ animals did not manifest enhanced mortality relative to WT controls. So while MBL-dependent mechanisms are involved host resistance against *T. cruzi*, other factors are clearly also important. Consistent with this, MyD88^−/−^ and bradykinin receptor^−/−^ mice infected with *T. cruzi* both display impaired IL-12/23p40 and IFN-γ production, as well as increased mortality [17], [32]. Thus, MBL-dependent regulation of IL-12/23p40 production is likely dispensable in the presence of other innate recognition systems such as TLRs and bradykinin, which are sufficient for mounting a protective Th1 response. This in turn is supported by our observation of similar systemic levels of IFN-γ between *T. cruzi*-infected WT and MBL^−/−^ mice.

The inflammatory response that follows *T. cruzi* infection is important for host resistance but also contributes to the pathology observed in Chagas’ disease [33]. A role for MBL as a modulator of inflammatory responses and cardiovascular disease is becoming increasingly evident. A correlation between increased MBL levels and coronary artery disease and myocardial infarction exists in men [34]. A role for MBL has also been demonstrated in ruptured atherosclerotic plaques [35]. This is at variance with our observations in *T. cruzi* where cardiac pathology is increased in the absence of MBL. Increased tissue damage observed in our model could thus be a consequence of increased parasite replication *in situ* in the absence of MBL. Corroborating this, some MBL-deficient patients have an increased risk of dying from infective endocarditis, likely caused by unchecked host control of *S. aureus* [36].

Increased *T. cruzi* loads in MBL^−/−^ mice are strongly in accord with recent genetic studies showing that low-responser MBL and MASP-2 genotypes in humans are overrepresented among patients with Chagas’ disease [22], [23] and Chagas’ cardiomyopathy [22]. The exact mechanism by which MBL regulates host control of *T. cruzi* growth was however not formally addressed in this study. Because the surface of trypomastigotes contains several molecules capable of interfering with the actions of complement [20], [37], [38], direct complement-mediated killing of trypomastigotes seems unlikely. This is the case even though the lectin pathway of complement activation is only partially ablated in the absence of MBL, due to the presence of ficolins [39]. On the other hand, the intracellular amastigote form of *T. cruzi* is highly sensitive to complement [19] and MBL has been shown to bind to amastigote forms of *Trypanosoma* [40] as well as *Leishmania* [41]. Interestingly, amastigotes can be released together with trypomastigotes when infected host cells rupture [42], [43]. These amastigotes are infectious and capable of sustaining the *T. cruzi* life cycle [42], [43], [44]. Thus, impaired complement-mediated clearance of amastigotes in the absence of MBL would generate an additional source of infectious organisms in the host, leading to an overall increase in parasite burden in MBL^−/−^ mice. In fact, progressive accumulation of amastigote-derived antigens and pro-inflammatory molecules has been implicated in the immunopathology of Chagas’ disease [45], thus corroborating our finding of enhanced fibrosing myocarditis in MBL^−/−^ mice. Since it remains unknown if ficolins bind amastigotes, and whether the phenotype of MBL^−/−^ mice reflects an actual deficiency in complement activation, it would be interesting in future studies to investigate the outcome of *T. cruzi* infection in MASP-2^−/−^ mice [46], which are entirely deficient in the lectin pathway of complement activation.

We have demonstrated that MBL expression is also enhanced in acutely *T. cruzi*-infected, symptomatic patients. Our data supports recent reports showing that SNPs in the human MBL gene may be associated with susceptibility to *T. cruzi* [22], [23]. Collectively, these observations highlight the need for confirmatory experiments on the expression of MBL in additional and larger patient cohorts with Chagas’ disease. Genetic variations in both MBL and ficolin-2 have recently been associated with both visceral and cutaneous leishmaniasis [47], [48]. Thus, it will be important to follow up our observations made in *T. cruzi*-infected mice with robust clinical studies of MBL polymorphisms and clinical endpoints in Chagas’ disease patients. Clinical investigations together with continued dissection of MBL-regulated cardiac pathology in the mouse model of infection will surely lead to an enhanced understanding of the role played by MBL in the outcome of *T. cruzi* infection.

## Materials and Methods

### Mice

#### Ethics statement

This study was performed in accordance with the recommendations in the Guide for the Care and Use of Laboratory Animals of the NIH (Bethesda, MD) and AVMA guidelines for Euthanasia. Animals were bred and maintained under specific pathogen-free conditions at an AALAC-accredited facility at the NIAID under the animal-study proposal LPD14E, approved by the Institutional Animal Care and Use Committee (IACUC) of the NIH. All efforts were made to minimize suffering.

Mice double deficient in MBL-A and MBL-C (MBL^−/−^) have been previously described [5] as have MyD88^−/−^ mice [49]. MBL-deficient animals were backcrossed onto a C57Bl/6 background for 10 generations. MBL deficiency was confirmed by PCR (data not shown). C57Bl/6 and Swiss Webster mice were purchased from Taconic (Taconic Farms, Germantown, NY). C57Bl/6 mice were used as wild-type (WT) controls. Sex-matched animals between 8 and 12 wk old were used in all experiments.

### Patients

#### Ethics statement

All patients and control subjects have been informed of the study and have given written consent for blood sampling. The UFSC Medical Ethics Committees have approved the study protocol (UFSC- 144/2004).

Sera from symptomatic patients diagnosed with chronic (n = 10) or acute (n = 10) Chagas’ disease were selected. AIDS, diabetes, hepatitis, hypertension, pregnancy, and alcoholism were exclusion criteria. All patients included in the study have been confirmed by parasitological, serological and PCR assays for *T. cruzi* infection. Ten indeterminate asymptomatic *T. cruzi*-infected individuals were included in the control group.

### Parasites and Experimental Infection with *T. cruzi*

Blood-stage trypomastigote forms of the Y [50] and Colombiana [27] strains of *Trypanosoma cruzi*, both kindly obtained from Drs. Fuyuki Tokumasu (Laboratory of Malaria and Vector Research, NIAID/NIH), were maintained by serial passages in Swiss Webster outbred mice. For infections, mice were intraperitoneally inoculated with 1000 trypomastigotes of the Y or Colombiana strain of parasites. Parasitemia was determined using 5 µl of whole blood drawn from the tail vein by Brener’s method [51].

### Serum Cytokine and MBL Measurements

IFN-γ and IL-12/23p40 were measured in the serum of naïve and *T. cruzi*-infected mice by sandwich ELISA as previously described [52]. MBL was quantified in the serum of Chagas’ disease patients by ELISA (Hycult, HK323) following the manufacturer’s protocol.

### Gene Expression Measurement by Real-time PCR

Total RNA was isolated from infected and naïve heart using the RNeasy kit (Qiagen, Valencia, CA) according to the instructions of the manufacturer and 1 µg of total RNA reverse-transcribed into cDNA using M-MLV reverse transcriptase (Promega, Madison, WI). Real-time PCR was performed on an ABI Prism 7900 sequence detection system (Applied Biosystems, Foster City, CA) using SYBR Green PCR Master Mix. The relative amount of gene expression was determined by the comparative threshold cycle method as described by the manufacturer, whereby data for each sample were normalized to hypoxanthine phosphoribosyltransferase (HPRT) and the gene of interest expressed either as a fold change compared to uninfected controls or presented without normalization (arbitrary units). The following primer pairs were used:

for HPRT, GTT GGT TAC AGG CCA GAC TTT GTT G (forward) and

GAG GGT AGG CTG GCC TAT AGG CT (reverse);

for MBL-A, GCT CCT TTA CTC TAA AGA AAC CCT AGT (forward) and

TCA CCA CAC ACA GAA GGA CAG (reverse);

for MBL-C, AGG GAG AAA AGG GAG AAC CA (forward) and

CCT GGG GGT CCT GTA GGT (reverse);

for Collagen 1, AAC TGG ACT GTC CCA ACC CC (forward) and

TCC CTC GAC TCC TAC ATC TTC TG (reverse);

for Collagen 3, AAC CTG GTT TCT TCT CAC CC TTC (forward) and

ACT CAT AGG ACT GAC CAA GGT GG (reverse);

for Collagen 6, CGC CCT TCC CAC TGA CAA (forward) and

GCG TTC CCT TTA AGA CAG TTG AG (reverse).

### Quantification of Parasite Tissue Loads by Real-time PCR

Real-time PCR for parasite quantification was performed as previously described [53], with minor modifications. Briefly, at different time-points after infection, DNA was extracted from heart or spleen using a DNeasy kit (Qiagen). Real-time PCR using 50 ng of total DNA was performed on an ABI PRISM 7900 sequence detection system (Applied Biosystems) using SYBR Green PCR Master Mix according to the manufacturer’s recommendations. The equivalence of host DNA in the samples was confirmed by measurement of genomic IL-12/23p40 PCR product levels in the same samples. Purified *T. cruzi* DNA, obtained from trypomastigote cultures maintained *in vitro*, was sequentially diluted for standard curve generation in aqueous solution containing equivalent amounts of DNA from uninfected mouse tissues. The following primers were used: for *T. cruzi* minicircle specific-primers, GCT CTT GCC CAC AMG GGT GC, where M = A or C (S35-forward) and CCA AGC AGC GGA TAG TTC AGG (S36-reverse) for genomic IL-12/23p40, GTA GAG GTG GAC TGG ACT CC (forward) and CAG ATG TGA GTG GCT CAG AG (reverse).

### Histopathology

Hearts from WT and MBL^−/−^ mice infected with Colombiana strain of *T. cruzi* were harvested 5 wks after infection and fixed in 10% neutral-buffered formalin. Hearts were then paraffin-embedded, sectioned and stained with hematoxylin/eosin (H&E), picro-sirius red or Gomori’s Trichrome (Histoserv, Germantown, MD). Slides were examined under double-blinded conditions by a pathologist (A. W. C). Infiltrates were graded 1 to 4+on the basis of the density of cells and extent of the infiltrate. A single row of inflammatory cells was rated 1+and clusters of 3- to 4-cell thick and involving 10% of the myocardial area were graded 4+. Infiltrates extending into the myocardium from the epicardial and endocardial infiltrates were graded as part of the latter infiltrates. Interstitial fibrosis between myocardial fibers based on picro-sirius or Gomori’s Trichrome stainings were also graded 1 to 4+.

### Hydroxyproline Measurements

Hearts from WT and MBL^−/−^ mice infected with Colombiana strain of *T. cruzi* were obtained 5 weeks after infection and used to estimate collagen as hydroxyproline by method B of Bergman and Loxley as previously described [54].

### Immunofluorescence Microscopy

Spleens were excised from naïve and Y-strain infected animals, fixed overnight with 4% paraformaldehyde/PBS followed by dehydration in 30% sucrose/PBS prior to embedding in Tissue-Tek OCT freezing media (Sakura Finetek, Torrance, CA). Twenty micron-thick sections were cut on a CM3050s cryostat (Leica Microsystems, Bannockburn, IL) and adhered to Superfrost Plus slides (VWR, West Chester, PA). Sections were permeabilized and blocked in PBS containing 0.3% Triton X-100 (Sigma) and 10% donkey serum (Jackson Immunoresearch). This was followed by overnight incubation at 4°C with AlexaFluor 488-conjugated rat anti-CD4 (RM4-5), AlexaFluor 647-conjugated rat anti-B220 (RA3-6B2, both from BD Biosciences, San Diego, CA) and unconjugated, polyclonal goat anti-MBL-C (Santa Cruz Biotechnology, Santa Cruz, CA). Unconjugated anti-MBL-C was detected by staining with AlexaFluor 546-conjugated donkey anti-goat secondary antibodies (Invitrogen). Slides were counterstained with Hoechst 33342 and mounted with Prolong Gold (both from Invitrogen). 3D image stacks of splenic sections were acquired on a SP5 confocal microscope (Leica Microsystems). Images are displayed as 2D maximum intensity projections.

### Statistical Analyses

The significance of differences in data group means was analyzed by Student’s *t* test or non-parametric Mann Whitney test with a cut-off of p<0.05.
